# Multimodality Imaging–Guided Transseptal Closure of an Iatrogenic Left Ventricular Pseudoaneurysm

**DOI:** 10.1016/j.jaccas.2025.106210

**Published:** 2025-12-17

**Authors:** Serigne Cheikh Tidiane Ndao, Mauro Boiago, Riccardo Mager, Annabelle Nguyen, Gaetano Liccardo, Myriam Akodad, Bernard Chevalier, Pierre Demondion, Jérôme Garot, Philippe Garot

**Affiliations:** Institut Cardiovasculaire Paris Sud, Hôpital Privé Jacques-Cartier, Massy, France

**Keywords:** aortic valve, percutaneous coronary intervention, valve repair

## Abstract

**Background:**

Left ventricular (LV) pseudoaneurysm is a rare but potentially fatal complication, typically resulting from a contained rupture of the LV free wall after myocardial infarction or cardiac surgery.

**Case Summary:**

We report the case of a 65-year-old woman in whom a giant LV pseudoaneurysm was incidentally identified, likely as a late consequence of pericardiocentesis. Multimodality imaging, including cardiac computed tomography and transesophageal echocardiography, was essential for diagnosis and procedural planning. Given the patient's comorbidities and anatomic suitability, the heart team opted for percutaneous closure. A transseptal approach was used, with real-time computed tomography fluoroscopy fusion imaging to guide deployment of an Amplatzer muscular ventricular septal defect occluder. The intervention was successful, achieving complete exclusion of the pseudoaneurysm.

**Discussion:**

This case illustrates the feasibility and safety of transcatheter closure in high-risk patients.

**Take-Home Message:**

Percutaneous closure guided by fusion imaging is a viable alternative for managing LV pseudoaneurysms in high-risk surgical candidates.

## Central Illustration

**Figure:**
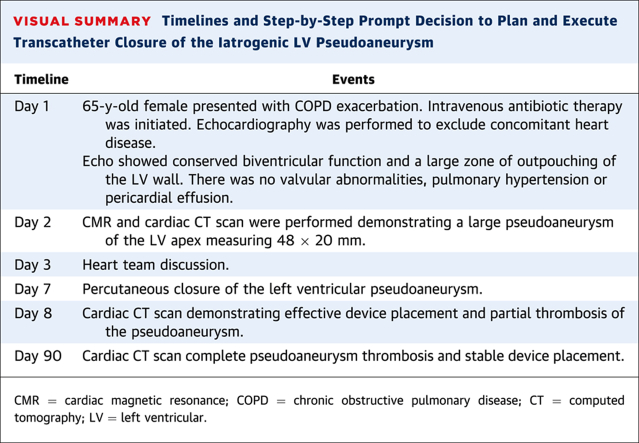

## History of Presentation

**Figure 1:**
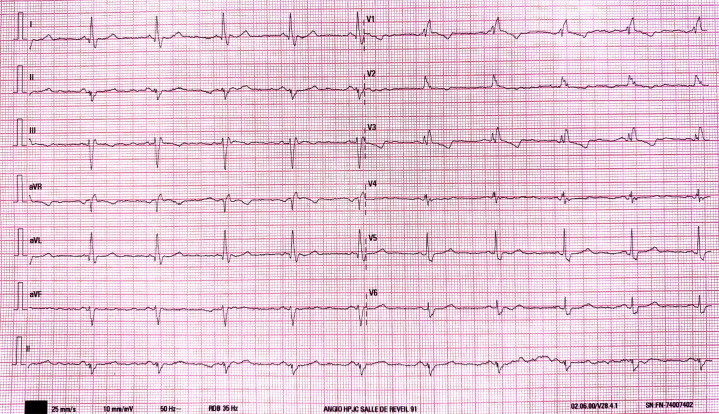
ECG at Admission The ECG showed a sinus rhythm with complete right bundle branch block and left anterior hemiblock.

A 65-year-old woman was admitted to the internal medicine department for severe exacerbation of chronic obstructive pulmonary disease. On admission, empirical intravenous antibiotic therapy was initiated, resulting in rapid clinical improvement. During hospitalization, ECG showed complete RBBB and LAHB (Figure 1), and transthoracic echocardiography revealed a large outpouching of the left ventricular (LV) wall with disturbed flow at the apex along with a normal ejection fraction and wall motion. She was subsequently transferred to the cardiology department for further evaluation.

**Take-Home Messages**

• Percutaneous pericardiocentesis via apical or parasternal approach should be avoided, when possible, due to risks such as pneumothorax and myocardial injury.
• The subxiphoid route under imaging guidance remains the standard of care.
• In selected high-risk patients, percutaneous closure of LV pseudoaneurysms guided by TEE and CCT-fluoroscopy fusion is a safe and effective alternative to surgery.

## Past Medical History

The patient’s medical history included former tobacco use, chronic obstructive pulmonary disease, and lung cancer treated with chemotherapy 2 years earlier. During chemotherapy, she developed a significant pericardial effusion. A percutaneous drainage attempt via a left parasternal approach was initially unsuccessful, necessitating surgical evacuation. Cytologic and histologic analysis revealed no malignant cells. The patient completed cancer treatment without recurrence, and her tumor was considered in remission.

## Differential Diagnosis

The findings were consistent with either a true LV aneurysm or a pseudoaneurysm (PA).

## Investigations

**Figure 2:**
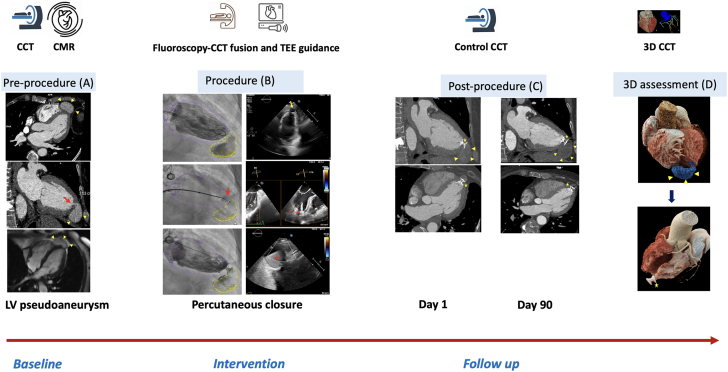
Multimodality Cardiac Imaging to Plan, Guide, and Assess the Result of Transcatheter Closure of an Iatrogenic Left Ventricular Pseudoaneurysm (A) Preprocedure: multimodality imaging (CCT and CMR) identifies a large LV pseudoaneurysm (yellow arrowheads) with a narrow neck (red arrow), nonthrombosed and lined by a thin tissue layer. (B) Procedure: CCT–fluoroscopy fusion with real-time TEE guidance. The ventricular septal defect (VSD) device is positioned across the neck (red arrow), initiating partial thrombosis of the cavity (red star). (C) Follow-up: at 1 and 90 days, imaging confirms complete thrombosis of the pseudoaneurysm (yellow arrowheads) with stable device position (yellow star) sealing the neck. (D) 3D CCT reconstruction: preprocedural pseudoaneurysm (blue and yellow arrowheads) fully excluded postprocedure, with the VSD device in place (yellow star). 3D = 3-dimensional; CCT = cardiac computed tomography; CMR = cardiac magnetic resonance; LV = left ventricular; TEE = transesophageal echocardiography.

To further characterize the abnormality identified by echocardiography, cross-sectional imaging was performed. Cardiac magnetic resonance and cardiac computed tomography (CCT) confirmed the presence of a large apical LV PA measuring 48 × 20 mm, with an 8- × 5-mm neck communicating with the LV cavity and no evidence of thrombus (Figure 2A).

The case was reviewed by the heart team. In the absence of any cardiovascular history suggestive of a potential etiology—particularly myocardial infarction with no evidence of infarction on cardiac magnetic resonance—or prior cardiac surgery, the PA was suspected to be a late complication of the prior pericardiocentesis. Although the patient was asymptomatic and the PA was discovered incidentally, its substantial size posed a high risk of sudden rupture and could predispose to heart failure and peripheral thromboembolic events. Given the patient's comorbidities, percutaneous closure was deemed anatomically feasible and preferable to surgery.

## Management

In the patient under general anesthesia, right femoral vein access was obtained under ultrasound guidance, followed by preclosure using a single Perclose ProGlide device (Abbott Medical). Transseptal puncture was performed under transesophageal echocardiographic guidance using a BRK needle and Mullins dilator (Abbott Medical). Intravenous heparin was administered (100 IU/kg) to maintain an activated clotting time >250 seconds.

A FlexCath Steerable Sheath (Medtronic Inc) was advanced into the left atrium over a stiff.

An Amplatzer guidewire (Abbott Medical) was used to facilitate catheter manipulation toward the LV apex. Initial ventriculography was performed via a 5-F pigtail catheter (Cordis), which was subsequently exchanged for a 5-F multipurpose catheter. Under CCT-fluoroscopy fusion imaging guidance (Video 1), the multipurpose catheter was used to engage the PA cavity.

A 6-F, 110-cm Flexor introducer (Medtronic Inc) was then advanced over a 0.035-in J-tip guidewire. An 8-mm Amplatzer muscular ventricular septal defect (VSD) occluder (Abbott) was delivered through the introducer and deployed with the distal disk positioned within the PA, the proximal disk in the LV cavity, and the waist spanning the defect (Video 2).

Device stability was confirmed through gentle traction maneuvers before final release.

Repeat ventriculography demonstrated successful exclusion of the PA, with only minimal residual contrast staining in the cavity (Video 3). Transesophageal echocardiography (TEE) confirmed partial thrombosis of the PA (Figure 2B). Total procedural time was 73 minutes, with 20 minutes of fluoroscopy and 90 mL of contrast used. Radiation exposure and dose-area product were 239.41 mGy and 22.06 Gy cm^2^, respectively.

## Outcome and Follow-Up

CCT performed the following day confirmed effective exclusion of the PA (Figure 2C). The patient experienced no procedural complications and was discharged on postoperative day 2.

At 3 months, follow-up CCT showed complete closure of the PA without residual flow (Figure 2C).

## Discussion

LV PA is a rare but potentially fatal condition resulting from rupture of the LV free wall contained by adherent pericardium. Complications include rupture, heart failure, and peripheral embolism. Unlike true aneurysms, where the ventricular wall is thinned and dyskinetic yet intact, PAs have a disrupted myocardial wall and carry a markedly higher risk of rupture, with mortality rates reported between 30% and 45%.^1^^,^^2^

Most LV PAs result from myocardial infarction (55%), followed by cardiac surgery, trauma, and infection.^3^ PA after percutaneous pericardiocentesis is uncommon. When performed by skilled operators via the subxiphoid approach under echocardiographic or fluoroscopic guidance, this procedure is generally considered safe.^4^^,^^5^ However, alternative routes such as apical or parasternal approaches (as used in this case) can increase the risk of complications, including pneumothorax and direct myocardial injury.

Multimodality imaging plays a central role not only in diagnosis but also in procedural planning.^6^^,^^7^ CCT was essential for accurately identifying and sizing both the apical VSD and the PA, and assessing their anatomic relationship with surrounding structures. Measurements derived from CCT were used to size the VSD occluder, anticipating a 30% compression of the device by the LV wall. Fluoroscopy and TEE played a crucial role in procedure execution. Fluoroscopy, guided by CCT fusion, facilitated the crossing of the PA neck using a 0.035-in guidewire. This hybrid imaging approach proved particularly valuable because the PA is not visible under fluoroscopy alone, and TEE is not suitable for guiding the passage of a wire through a very small VSD. Fusion imaging enabled accurate localization of the PA neck, allowing for efficient and safe wire crossing, thereby minimizing procedural risk. The VSD occluder was then deployed under fluoroscopic guidance. The distal disk was carefully deployed within the PA cavity, followed by gentle traction of the delivery system toward the LV apex to secure proper positioning. The proximal disk was then deployed within the LV cavity, ensuring occlusion of the PA neck and stable anchoring of the device. TEE provided critical intraprocedural assessment during device deployment. Transseptal puncture was performed under TEE guidance, targeting a superior and mid anteroposterior location. This orientation was chosen to achieve optimal alignment with the mitral valve and subvalvular apparatus, facilitating a coaxial trajectory toward the LV apex. In addition, TEE confirmed the positioning of the occluder at the PA neck and allowed visualization of the waist compression, indicating effective sealing. A tug test was performed under TEE guidance to confirm device stability, showing no displacement despite gentle traction of the system within the LV cavity.

Although the treatment of acute or symptomatic PAs is well established, indications for intervention in chronic, asymptomatic cases remain debated.^8^ Nevertheless, it is reasonable to consider closure in PAs >3 cm or showing signs of expansion. Surgical repair remains the standard treatment; however, mortality can exceed 20% even in elective settings.^9^ Experience with percutaneous closure remains limited to case reports and small series.^10^ However, advancements in catheter techniques and occlusion devices have made transcatheter approaches increasingly viable, particularly for patients with prohibitive surgical risk.

The access route should be tailored to the anatomy and location of the PA. Although retrograde arterial access is commonly used for basal or posteroinferior defects, we selected a transseptal approach to optimize coaxial alignment with the apical PA. Steerable sheaths provided enhanced control and facilitated device deployment.

PAs with a small pouch and a narrow neck may be treated with coils. In most other cases, closure requires deployment of an occluder device. A muscular VSD occluder was selected over other device types, such as atrial septal defect or patent ductus arteriosus occluders, to avoid mechanical interference with the left ventricle during systole or a higher risk of embolization. Device type (atrial vs ventricular) and size should be guided by preprocedural imaging. In this case, an 8-mm Amplatzer muscular VSD occluder was selected based on CCT measurements with an anticipated device compression of approximately 30%, which we judged to be optimal for secure deployment and stability.

This case also illustrates the value of fusion imaging (CCT-fluoroscopy) in enhancing safety and precision, while reducing contrast volume, radiation exposure, and procedural duration.

## Conclusions

We report a successful percutaneous closure of a large, iatrogenic apical LV PA using a muscular VSD occluder, guided by TEE and CCT-fluoroscopy fusion imaging, in a patient with elevated surgical risk.

## Funding Support and Author Disclosures

Dr Garot is a proctor and consultant for Abbott. All other authors have reported that they have no relationships relevant to the contents of this paper to disclose.

**Visual Summary:** Timelines and Step-by-Step Prompt Decision to Plan and Execute Transcatheter Closure of the Iatrogenic LV Pseudoaneurysm

Timeline	Events
Day 1	65-y-old female presented with COPD exacerbation. Intravenous antibiotic therapy was initiated. Echocardiography was performed to exclude concomitant heart disease. Echo showed conserved biventricular function and a large zone of outpouching of the LV wall. There was no valvular abnormalities, pulmonary hypertension or pericardial effusion.
Day 2	CMR and cardiac CT scan were performed demonstrating a large pseudoaneurysm of the LV apex measuring 48 × 20 mm.
Day 3	Heart team discussion.
Day 7	Percutaneous closure of the left ventricular pseudoaneurysm.
Day 8	Cardiac CT scan demonstrating effective device placement and partial thrombosis of the pseudoaneurysm.
Day 90	Cardiac CT scan complete pseudoaneurysm thrombosis and stable device placement.

CMR = cardiac magnetic resonance; COPD = chronic obstructive pulmonary disease; CT = computed tomography; LV = left ventricular.

**Equipment List:** 

Access • 0.035-in J-wire (Cordis SA) • 6-F femoral sheath (Terumo Europe) • 1 Proglide (Abbott Vascular) Transseptal puncture and LV access • 0.032-in J-wire • Flexor Introducer 6/110 (Cook) • Swartz transseptal introducer 8.5 (Abbott Medical) • BRK 45° transseptal needle (Abbott Medical) • FlexCath Steerable Sheath (Medtronic) • Impulse 5-F Pigtail (Boston Scientific) • 5-F multipurpose catheter (Cordis SA) • Stiff Amplatzer guidewire (Abbott Medical) • Amplatzer TorqVue Delivery System 7-F 80-cm 45°(Abbott Medical) LV pseudoaneurysm closure device • Amplatzer Muscular VSD occluder 8/7 (Aga Medical)

LV = left ventricular.

## Appendix

Video 1 Cardiac Computed Tomography–Fluoroscopy Fusion Imaging Guidance Ventriculography shows clearly the left ventricular cavity, the pseudoaneurysm, and its neck before engagement by the multipurpose catheter.
